# Impact of zinc deficiency on the recurrence in aphthous stomatitis: A prospective case-control study

**DOI:** 10.6026/973206300223599

**Published:** 2026-06-30

**Authors:** Ashutosh Pandey, Ganiga Channaiah Shivakumar, Kavita Kumari Anshu, Anjana Bharti, Sumit Verma, Sagar Mani, Palak Jain Choudhary

**Affiliations:** 1 Department of Dentistry, Anugrah Narayan Magadh Medical College and Hospital, Gaya, Bihar, India; 2 Department of Oral Medicine and Radiology, People's College of Dental Sciences and Research Centre, People's University, Bhopal, Madhya Pradesh, India; 3 Department of Public Health Dentistry, Mithila Minority Dental College and Hospital, Darbhanga, Bihar, India; 4 Department of Oral and Maxillofacial Surgery, Buddha Institute of Dental Sciences and Hospital, Patna, Bihar, IndiaDepartment of Oral and Maxillofacial Surgery, Buddha Institute of Dental Sciences and Hospital, Patna, Bihar, India; 5 Department of Oral Medicine and Radiology, Private Practitioner, Your Dentist, Jhumri Telaiya, Jharkhand, India

**Keywords:** Stomatitis, aphthous, dietary supplements, recurrence, quality of life, zinc

## Abstract

Recurrent aphthous stomatitis (RAS) is one of the frequent problems of the mouth that affects 10 to 20 percent of the population. Therefore, it is of interest to find out if the level of zinc in the blood is related to the recurrence of RAS and if taking zinc can reduce the recurrence and severity of RAS. We enrolled 30 RAS patients with minor recurrent aphthous stomatitis, and 30 healthy controls matched to the RAS patients by age and sex from the clinic over 12 months and RAS patients had significantly lower fasting serum zinc levels than the controls (76.4 ± 11.2 µg/dL vs. 87.3 ±8.9 µg/dL; p < 0.001). Zinc deficiency was seen in 56.7 percent of RAS patients with serum zinc levels less than 70 µg/dL compared with 16.7 percent of the controls (p < 0.001). Thus, low serum levels of zinc are significant predictors of recurrence of RAS and zinc can be used as an additional preventive measure against RAS.

## Background:

One of the most common oral disorders encountered in day-to-day dental practice is recurrent aphthous stomatitis (RAS) [1]. RAS is a chronic , relapsing and inflammatory disorder and the exact etiology remains a mystery [2]. RAS has been clinically characterized as recurring, discrete, painful ulcers, which are usually oval in shape, have a reddish border and a yellowish base [3]. Several theories have been proposed regarding the etiology of RAS, but the strongest evidence supports the theory of an abnormal immune response mediated by T-cells in individuals with genetic predisposition, which is initiated by various factors related to health and health status in general [4]. Deficiency in nutrients such as vitamin B12, folate, iron and trace elements has been associated with increased risk of oral ulcers [5 , 6]. Zinc is an essential trace mineral required for the proper functioning of more than 300 enzymes, which are responsible for various cellular activities such as growth, differentiation and immunity, both cellular and humoral [7]. Zinc has been known to play an important role in the oral cavity, including cellular turnover, collagen linking and healing [8]. Although it is logical that zinc has a role in RAS, initial clinical studies regarding zinc and RAS have been inconclusive regarding zinc deficiency as a causative factor for RAS recurrence [9 , 10]. RAS has a significant effect on the quality of life of those suffering from it, as the painful ulcers make it difficult for them to eat, speak and maintain oral hygiene [11]. The available treatments for RAS are symptomatic, and no attempt is made to correct nutritional deficiencies, which are thought to be responsible for RAS recurrence [12]. Therefore, it is of interest to determine whether a correlation exists between RAS recurrence and zinc levels in the blood and whether zinc supplementation can prevent RAS recurrence in zinc-deficient patients.

## Materials and Methods:

## Study design and ethical considerations:

The study was a prospective case-control study, conducted over 12 months at a tertiary care dental center. Ethical clearance was obtained before the study and an explanation of the study, along with the risks, was given to the patients, followed by their consent to participate in the study. The study was conducted according to the principles of the Declaration of Helsinki.

## Participants and grouping:

A total of 30 patients with a confirmed diagnosis of minor type RAS were included in the study as the case group, along with 30 patients without a history of oral ulcers as the control group, matching the case group in terms of age and sex. The patients with minor type RAS were selected if they had at least three episodes of RAS in the past year with an active ulcer at the time of screening. The patients were excluded if they had underlying conditions that cause similar types of oral ulcers, such as Behcets disease, celiac disease, Crohns disease; if they were on immunosuppressants, nutritional supplements, pregnant, or lactating.

## Baseline clinical assessment:

For every patient, the following information was recorded at the time of screening: the age at first onset of RAS, the family history of RAS and the frequency of RAS per year. The size of the largest ulcer was measured with the help of a calibrated probe, while the pain was measured with the help of a 10-cm Visual Analogue Scale, ranging from 0 mm, no pain, to 100 mm, maximum pain. The oral health-related quality of life was measured by the 14-item OHIP-14.

## Biochemical evaluation:

Five millilitres of venous blood was collected in the morning after fasting the previous night to avoid the diurnal variation of zinc levels. Serum was separated and stored at -20°C until analysis. Flame atomic absorption spectroscopy was used to determine the level of zinc in the serum. Patients with zinc deficiency were defined as having serum zinc concentrations below 70 µg/dL. The normal range of serum zinc was between 70 and 80 µg/dL.

## Supplementation protocol:

RAS patients with initial serum zinc levels at or below 80 µg/dL were enrolled in the study and received a three-month regimen of oral zinc gluconate, which contained 15 mg of elemental zinc daily. Patients with serum zinc levels below 80 µg/dL but did not wish to take the supplement were included in the unsupplemented group.

## Follow-Up and outcomes:

Patients returned at one-month intervals over the course of the three-month study. The outcomes of interest were the change in serum zinc levels and the development of new aphthous ulcers. Additional outcomes included change in the size of the ulcers, time to heal, pain as measured by the visual analog scale (VAS) and OHIP-14 score. Adverse effects of the supplement were also monitored.

## Statistical methods:

Data analysis was done using SPSS version 25.0 software. Continuous variables are expressed as mean ± SD. Independent-samples t-test or Mann-Whitney U test was used to compare between groups and the paired-samples t-test was used to compare within groups. Correlation between variables was done using Pearson correlation coefficient. Significance was set at two-tailed p-value less than 0.05.

## Results:

Of the 65 individuals initially screened, 60 (30 cases and 30 controls) fulfilled all eligibility criteria and completed the full three -month protocol. The combined sample comprised 37 women (61.7%) and 23 men (38.3%), with a mean age of 38.2 ± 11.6 years (range 20-62 years). No statistically significant differences were noted between the two groups with respect to age, sex distribution, or socioeconomic background (all p > 0.05) (Table 1). Mean serum zinc was substantially lower among RAS patients (76.4 ± 11.2 µg/dL) than among controls (87.3 ± 8.9 µg/dL; p < 0.001). Overt zinc deficiency(<70 µg/dL) was documented in 17 of 30 RAS patients (56.7%), compared with 5 of 30 controls (16.7%; p < 0.001). Seven additional RAS patients (23.3%) had borderline zinc concentrations (70-80 µg/dL), a proportion that did not differ significantly from controls (8 of 30, 26.7%; p = 0.78). A statistically significant inverse correlation emerged between baseline serum zinc and the self-reported annual frequency of aphthous episodes (r = -0.612, p < 0.001), indicating that progressively lower zinc concentrations were accompanied by a greater recurrence burden. This pattern was further reflected in the markedly higher mean annual episode count among zinc-deficient RAS patients (7.2 ± 2.8) relative to those with normal zinc levels (3.1 ± 1.4; p < 0.001) (Table 2). Of the 24 RAS patients with deficient or borderline serum zinc (≤80 µg/dL), 17 consented to the supplementation regimen (15 mg elemental zinc/day for three months), whereas the remaining 7 declined and were retained as an unsupplemented observational subgroup. In the supplemented cohort, mean serum zinc rose from 69.2 ± 8.1 µg/dL at enrolment to 91.4 ± 7.3 µg/dL at the three-month assessment (p < 0.001), representing a 32.1% improvement. Concurrently, the mean monthly episode frequency declined from 0.63 ± 0.21 to 0.17 ± 0.12 in supplemented patients, representing a 72.7% reduction (p < 0.001). In the unsupplemented subgroup, the corresponding change was marginal (0.58 ± 0.19 to 0.51 ± 0.18; p = 0.28). By the final month of the intervention period, 14 of 17 supplemented patients (82.4%) reported complete cessation of new ulcer formation, as against only 1 of 7 unsupplemented patients (14.3%). Clinically, residual lesions in the supplemented group were smaller in diameter (4.2 ± 1.4 mm versus 6.8 ± 1.9 mm at baseline; p < 0.001), healed nearly twice as rapidly (5.1 ± 1.6 days versus 9.2 ± 2.1 days; p < 0.001) and were accompanied by substantially less pain (VAS 3.2 ± 1.4 versus 6.8 ± 1.7; p < 0.001). The unsupplemented observation group exhibited no meaningful change in any of these parameters over the same period (Table 3). Improvements in clinical indices were paralleled by patient-reported outcomes. In the supplemented subgroup, mean OHIP-14 scores decreased from 28.4 ± 8.2 at enrolment to 9.3 ± 5.1 at three months, representing a 67.2% improvement (p < 0.001). The unsupplemented subgroup showed no appreciable change over the same interval (28.1 ± 8.0 to 26.3 ± 7.9; p = 0.34) (Table 4). When the supplemented cohort was stratified according to the severity of baseline zinc depletion, patients with frank deficiency (<70 µg/dL) exhibited a markedly superior response compared with those in the low-normal range (70-80 µg/dL). Complete elimination of new episodes during the final month was achieved in 11 of 13 overtly deficient patients (84.6%), versus only 1 of 4 patients in the borderline category (25.0%; p < 0.05). Tolerability was favourable: only 2 of 17 supplemented patients (11.8%) reported transient gastrointestinal discomfort, which resolved upon ingesting the supplement with food. Self-reported adherence to the prescribed regimen was 94.1% (Table 5).

## Discussion:

This study offers clear evidence that zinc status is closely related to the frequency and severity of RAS. More than half (56.7%) of those with RAS were found to have clear zinc deficiencies. This is understandable in terms of the role zinc has in protecting the body, particularly in terms of the immune system, as well as the skin and mucous membranes [13, 14]. The level of zinc deficiencies was also in line with those found in RAS patients, which was around 70.8% and was greater than those found in another group [15], as measured by Ozler *et al.* [16], which was only 28%. A notable finding in this study was the clear relationship (r = -0.612) between zinc in the blood and the frequency of aphthous ulcers, suggesting that zinc status has a role in controlling RAS. Zinc is required for the production of various enzymes, particularly for the production of T-cells [17]. According to Haase and Rink [18] , zinc deficiencies lead to a decrease in the release of thymulin, affecting the growth of T-cells. This, in RAS, leads to an imbalance in the immune system, affecting the balance between Th1 and Th2 and leading to the inflammation in the mouth characteristic of RAS. The zinc supplementation was beneficial. The incidence of episodes decreased by 72.7% and this was over a period of three months of using zinc monthly. This is consistent with Yazdanpanah *et al.* [19], where zinc sulfate was beneficial for all zinc-deficient RAS patients. In addition, not only did the ulcers decrease in size, but also healing was twice as fast (5.1 days instead of 9.2 days). This is also consistent with Roohani *et al.* [20] where zinc was shown to be involved in tissue repair mechanisms. In addition, the patients' quality of life was enhanced. The OHIP-14 scores were reduced by 67.2%, indicating less pain during meals and increased confidence during social interactions. This is consistent with Krawiecka *et al.* [21], where chronic oral health disorders affect patients' quality of life. The more frequently and less severe ulcers occur, the more detrimental this is for the individual. This emphasizes the significance of zinc for reducing both the incidence and severity of RAS. Some research has shown that zinc does not help all RAS patients. This was shown in a study done by Wray *et al.* [22] , where zinc was not shown to be beneficial for RAS patients. However, their study was not based on zinc deficiency. In conclusion, zinc works best for zinc-deficient individuals. Zinc should not be given to all RAS patients without first checking zinc levels. The possible interactions of zinc with other nutrients that play an important role in tissue repair, such as vitamin B12 and folate, should be investigated further. The zinc dose of 15 mg/d was well tolerated and only 11.8% of patients had mild stomach discomfort, which was relieved by meals. This shows that zinc is safe for use. In addition, more than 94% of patients complied with zinc supplementation. This also shows that zinc is practical and safe for use. The positive sides of this study include that it has an open and long-term design that shows how things change over time. The use of flame atomic absorption spectroscopy ensured precise results for zinc levels. The use of both objective clinical measures and a validated patient-reported outcome measure (OHIP-14) provided an extensive understanding of the results. On the other hand, some of the limitations of this study include that it had a small sample size and did not use a double-blind placebo setup. This could have led to some biases. The study did not measure other nutrients such as vitamin B12, folate, or iron, which play an important role in the health of the mucosa. The study did not follow patients after three months of stopping supplementation and see if benefits last. These limitations show some of the future research directions that could be used. Future research should be conducted on how zinc works and how it could affect the immune system at a molecular level. Future research could also be conducted using topical zinc and how adding other nutrients could improve treatment for this common and painful condition.

## Conclusion:

We show that there is a clear and significant relationship between low levels of zinc and the recurrence of RAS. Giving zinc orally to zinc-deficient patients reduced how often RAS occurred,their size,healing time and pain and improved oral health-related quality of life by an enormous margin. This study suggests that serum zinc levels should be checked for patients with frequently recurring RAS as well as supports zinc supplementation as an additional, safe and accessible treatment for RAS.

## Figures and Tables

**Table 1:** Demographic characteristics of the study participants

**Characteristic**	**RAS Cases (n = 30)**	**Controls (n = 30)**	**p-value**
Age (years), mean ± SD	38.6 ± 12.1	37.8 ± 11.2	0.74
Sex (Male / Female)	19-Nov	18-Dec	0.81
SD = standard deviation.
p-values derived from
independent-samples
t-test (age)
and chi-square test (sex).

**Table 2:** Baseline serum zinc and clinical parameters in RAS patients versus controls

**Parameter**	**RAS Cases (n = 30)**	**Controls (n = 30)**	**p-value**
Mean serum zinc (µg/dL)	76.4 ± 11.2	87.3 ± 8.9	< 0.001*
Zinc deficiency (<70 µg/dL), n (%)	17 (56.7)	5 (16.7)	< 0.001*
Baseline pain intensity (VAS 0-10)	6.8 ± 1.7	0.2 ± 0.4	< 0.001*
*Statistically significant (p < 0.05).
VAS = Visual Analogue Scale.

**Table 3:** Effect of zinc supplementation on clinical parameters at baseline and three-month follow-up

**Parameter**	**Baseline**	**3-Month Follow-up**	**Change (%)**	**p-value**
Zinc-Supplemented				
Group (n = 17)				
Serum zinc (µg/dL)	69.2 ± 8.1	91.4 ± 7.3	32.1	< 0.001*
Monthly episode frequency	0.63 ± 0.21	0.17 ± 0.12	-72.7	< 0.001*
Lesion diameter (mm)	6.8 ± 1.9	4.2 ± 1.4	-38.2	< 0.001*
Healing time (days)	9.2 ± 2.1	5.1 ± 1.6	-44.5	< 0.001*
Pain intensity (VAS 0-10)	6.8 ± 1.7	3.2 ± 1.4	-52.9	< 0.001*
Unsupplemented Controls (n = 7)				
Serum zinc (µg/dL)	68.9 ± 8.4	70.1 ± 8.7	1.7	0.48
Monthly episode frequency	0.58 ± 0.19	0.51 ± 0.18	-12.1	0.28
Lesion diameter (mm)	7.1 ± 2.1	6.9 ± 2.0	-2.8	0.71
Healing time (days)	9.1 ± 2.3	8.9 ± 2.4	-2.2	0.64
Pain intensity (VAS 0-10)	6.9 ± 1.8	6.4 ± 1.9	-7.2	0.34
*Statistically significant
(p < 0.05). VAS = Visual Analogue Scale.
Values expressed as mean ± SD.

**Table 4:** Oral health-related quality of life (OHIP-14) at baseline and three-month follow-up

**Group**	**Baseline OHIP-14**	**3-Month OHIP-14**	**p-value**
Supplemented (n = 17)	28.4 ± 8.2	9.3 ± 5.1	< 0.001*
Unsupplemented (n = 7)	28.1 ± 8.0	26.3 ± 7.9	0.34
*Statistically significant
(p < 0.05). OHIP-14 = Oral Health Impact
Profile-14. Values expressed
as mean ± SD.

**Table 5:** Clinical response among supplemented patients stratified by baseline zinc status

**Parameter**	**Deficient (<70 µ g /dL)**	**Low-Normal (70-80 µ g /dL)**	**p-value**
Episode elimination, n (%)	11 / 13 (84.6)	1 / 4 (25.0)	< 0.05*
Pain reduction (%)	53.2 ±7.4	31.5 ±16.2	< 0.05*
*Statistically significant
(p < 0.05). Values
expressed as mean ±
SD where applicable.
